# Self‐awareness of acute post‐stroke cognitive decline

**DOI:** 10.1002/alz.093100

**Published:** 2025-01-09

**Authors:** Kim Caf, Martin Rakusa

**Affiliations:** ^1^ University Medical Centre Maribor, Maribor Slovenia

## Abstract

**Background:**

Post‐stroke cognitive impairment is a serious complication which could be easily overlooked. Our study aimed to evaluate patients' awareness of post‐stroke cognitive impairment.

**Methods:**

We included 74 patients (40 women) after acute stroke. Cognitive decline was assessed using the self‐reported questionnaire, the Montreal Cognitive Assessment (MoCA) and the Hachinski Ischaemic Score (HIS). A previously validated cut‐off score of 24/25 [1] was used to determine cognitive impairment. Patients were divided into four groups. Demographic data and MoCA score were compared among four groups using ANOVA.

**Results:**

The mean age of patients was 68.4 years (SD 14.32), the mean MoCA was 21.8 (SD 5.75), and the mean HIS was 8.1 (SD 2.8). Both self‐reported post‐stroke cognitive impairment incidence and MoCA‐evaluated incidence were 63%. MoCA score significantly correlated with education (r = 0.36; p<0.001) and age (r = ‐0.38; p<0.001), but not with HIS.

Thirty‐one patients were aware of their cognitive decline (MoCA 18.2; SD 1), 16 denied cognitive decline despite mean MoCA 20 (SD 1), 16 falsely reported cognitive decline (MoCA 27; SD 0.4), and 11 correctly denied cognitive impairment (MoCA 27, SD 0.4). There were no significant differences between groups in HIS.

For self‐reported post‐stroke cognitive impairment, sensitivity was 66%, specificity was 59%, positive predictive value was 66%, and negative predictive value was 59%. The accuracy rate was 57%, and precision 63%.

**Conclusions:**

our results demonstrate that cognitive impairment is common in patients after acute stroke. However, self‐awareness is low. Therefore, it is necessary to search for post‐stroke cognitive impairment actively, especially in older, lower‐educated patients.

**Reference**

[1] Potocnik J, Ovcar Stante K, Rakusa M (2020) The validity of the Montreal cognitive assessment (MoCA) for the screening of vascular cognitive impairment after ischemic stroke. Acta Neurol Belg 120:681‐685. https://doi.org/10.1007/s13760‐020‐01330‐5

